# Cost-Effectiveness of Adding Ribociclib to Endocrine Therapy for Patients With HR-Positive, HER2-Negative Advanced Breast Cancer Among Premenopausal or Perimenopausal Women

**DOI:** 10.3389/fonc.2021.658054

**Published:** 2021-05-07

**Authors:** Eunae Jeong, Changjun Wang, Leslie Wilson, Lixian Zhong

**Affiliations:** ^1^ Irma Lerma Rangel College of Pharmacy, Texas A&M University, College Station, TX, United States; ^2^ Department of Breast Surgery, Peking Union Medical College Hospital, Beijing, China; ^3^ Department of Clinical Pharmacy, School of Pharmacy, University of California, San Francisco, San Francisco, CA, United States

**Keywords:** breast cancer, CDK4/6 inhibitor, cost-effectiveness, partitioned survival analysis, ribociclib, pre/perimenopausal

## Abstract

**Purpose:**

To evaluate the cost-effectiveness of adding ribociclib to endocrine therapy for pre/perimenopausal women with hormone receptor-positive (HR+), human epidermal receptor 2-negative (HER2-) advanced breast cancer from the US payer perspective.

**Methods:**

A partitioned survival analysis model with three health states (progression-free, progressed disease, and death) was developed to compare the cost and effectiveness of ribociclib in combination with endocrine therapy versus endocrine therapy alone based on clinical data from the MONALEESA-7 phase 3 randomized clinical trials. Life years (LYs), quality-adjusted life-years (QALYs), and total costs were estimated and used to calculate incremental cost-effectiveness ratio (ICER) over a lifetime. Deterministic and probabilistic sensitivity analyses were conducted to test the uncertainties of model inputs. Additional scenario analyses were performed.

**Results:**

In the base-case, ribociclib plus endocrine therapy was more effective than endocrine therapy with an additional 1.39 QALYs but also more costly with an ICER of $282,996/QALY. One-way deterministic sensitivity analysis showed that overall survival associated with the treatments and the cost of ribociclib had the greatest impact on the ICER. The probabilistic sensitivity analysis showed that only beyond a willingness-to-pay (WTP) threshold of $272,867, ribociclib plus endocrine therapy would surpass endocrine therapy alone as a cost-effective option.

**Conclusions:**

From the US payer perspective, ribociclib plus endocrine therapy for pre/perimenopausal patients with HR+/HER2- advanced breast cancer is not cost-effective at a WTP threshold of $100,000 or $150,000 per QALY in comparison of endocrine therapy alone.

## Introduction

Breast cancer is the most common cancer in women worldwide and the second most common cancer among women in the United States (1, 2). The American Cancer Society projects that 276,000 new cases will be diagnosed, and about 42,000 women will die from breast cancer in 2020 (3). Approximately 30% of women diagnosed with early-stage breast cancer subsequently develop advanced or metastatic cancer (4). Currently, it is estimated that 155,000 women with metastatic breast cancer are living in the United States (5).

Cyclin-dependent kinase (CDK) 4/6 inhibitors in combination with endocrine therapy have been approved by the US Food and Drug Administration (FDA) for both first-line and second-line treatment of HR+/HER2- advanced or metastatic breast cancer (6–17).

Ribociclib (trade name: Kisqali^®^) is a CDK 4/6 inhibitor, approved by the FDA for postmenopausal women with HR+/HER2- advanced or metastatic breast cancer when used in combination with an aromatase inhibitor or with fulvestrant (18, 19). Ribociclib has also recently been approved for pre/peri menopausal women with HR+/HER2- advanced or metastatic breast cancer when used in combination with an aromatase inhibitor based on MONALEESA-7 (NCT02278120), a randomized, double-blind, placebo-controlled phase III trial (18, 19). In this pivotal trial, pre/perimenopausal women with advanced or metastatic breast cancer received endocrine therapy (tamoxifen or a non-steroidal aromatase inhibitor (NSAI)) either alone or in combination with ribociclib (9, 13). Ribociclib plus endocrine therapy significantly improved progression-free survival (PFS) (median PFS: 23.8 vs 13.0 months; hazard ratio (HR) 0.55; 95% confidence interval (CI) 0.44-0.69; p<0.0001) in comparison with endocrine therapy alone (placebo) (13). Additionally, the MONALEESA-7 clinical trial showed significantly prolonged overall survival (OS) for ribociclib plus endocrine therapy versus endocrine therapy alone (estimated OS rate at 42 months 70.2% (95% CI 63.5%-76.0%) vs. 46.0% (95% CI 32.0%-58.9%); death HR 0.71 (95% CI 0.54-0.95); p<0.01). MONALEESA-7 was the first trial to demonstrate a statistically significant overall survival benefit for a CDK 4/6 inhibitor plus endocrine therapy as the first-line treatment of advanced breast cancer. The most frequent grade 3 and 4 adverse events (AEs) for ribociclib compared to placebo were neutropenia (61% vs. 4%) and leukopenia (14% vs. 1%) (20).

While ribociclib has shown promising clinical effectiveness, it is associated with high cost. The wholesale acquisition cost (WAC) for ribociclib is $12,553 for a package of 63 200 mg tablets, which is the estimated amount needed for a 28-day treatment cycle. This study aims to evaluate the cost-effectiveness of ribociclib plus endocrine therapy versus endocrine therapy alone for pre/perimenopausal women with HR+/HER2- advanced breast cancer from a US payer perspective.

## Methods

### Patient Population

The model cohort characteristics were based on the patients enrolled in the MONALEESA-7 clinical trial (13, 20). The target population for the cost-effectiveness evaluation was pre/perimenopausal women aged between 18 and 59 who had histologically or cytologically confirmed HR+/HER2- advanced breast cancer. Patients who received endocrine therapy during advanced disease state or previous CDK4/6 inhibitor treatment were excluded.

### Intervention and Comparator

Treatment regimens in the economic analysis followed the MONALEESA-7 clinical trial protocol (20). The experimental intervention therapy was 600 mg of ribociclib (3×200 mg tablets) plus endocrine therapy administered orally once daily on day 1 to 21 in a 28-day cycle. Endocrine therapy alone without ribociclib was used as the comparator. Tamoxifen (20 mg orally once daily) or NSAI (letrozole 2.5 mg orally once daily or anastrozole (1 mg orally once daily) was used for endocrine therapy. For ovarian suppression treatment, goserelin 3.6 mg was administered subcutaneously on day 1 of the 28-day cycle (20). For both treatment and comparator groups, treatment was discontinued once the disease progressed (20).

### Partitioned Survival Analysis Model

#### Model Structure

**Figure 1:**
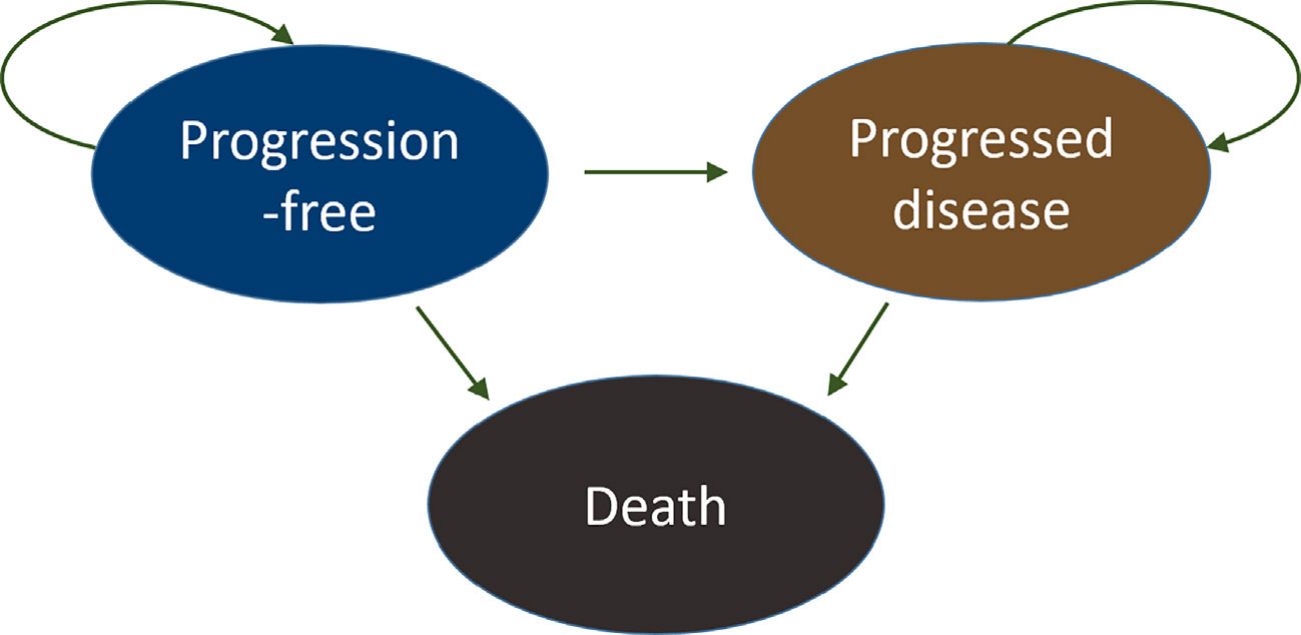
Partitioned survival analysis and the state diagram. Three health states were considered in the model: progression-free (PF), progressed disease (PD), and death (D). All patients started in the progression-free state and transitioned over time to progressed disease or death. Eventually, all subjects moved to the absorbing state, the death state. The arrows illustrate the directions of movements between different health states.

A cohort-based partitioned survival analysis (PartSA) model was constructed from a US payer perspective with three health states, progression-free (PF), progressed disease (PD), and death (D), to reflect the natural history of the disease and be consistent with clinical trial endpoints (**Figure 1**).

**Figure 2:**
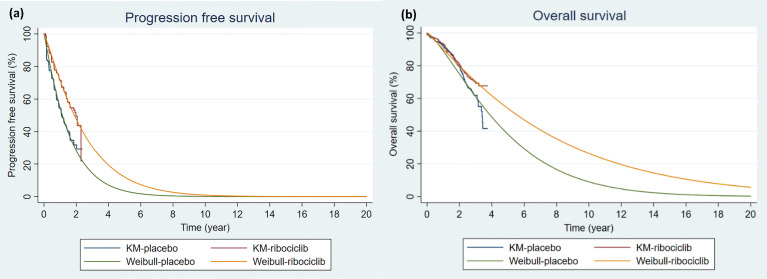
Kaplan-Meier Survival Curves and Parametric Survival Curve Fitting. **(A)** Progression-free survival **(B)** Overall survival. The Kaplan-Meier curves were constructed based on MONALEESA-7 clinical trial (13, 20). Weibull parametric distributions were fitted to the Kaplan-Meier curves for progression-free survival and overall survival over a 20-year time horizon. KM, Kaplan-Meier survival curve; placebo: endocrine therapy only; ribociclib, ribociclib and endocrine therapy; Weibull, extrapolated Weibull parametric model.

In this model, state occupancy of the cumulative probability of progression is estimated based on survival functions fitted to the original survival data. The PFS curve and the OS curve were obtained from the MONALEESA-7 clinical trial (13, 20). The web-based program WebPlotDigitizer was used to extract the PFS and OS from published Kaplan-Meier curves (21). The use of a fitted parametric distribution is generally preferred over using raw survival data in cost-effectiveness studies when the study time horizons are much longer than the study period. The extracted PFS and OS data were fitted by Exponential, Weibull, Gamma, Gompertz, Log-normal, and Log-logistic parametric distributions. Weibull parametric distribution, as illustrated in **Figure 2**, was chosen based on Akaike information criterion (AIC), Bayesian information criterion (BIC), and clinical relevance (22). The shape and scale parameters that determined the fitted Weibull distributions for PFS and OS for both arms were then used in the PartSA model, which mirrored disease progression by using the estimated state membership based on survival functions fitted to the PFS and OS data. The parametric survival functions obtained from the Kaplan-Meier curves of PFS and OS were provided for three health states: PF, PD, and D. The area under the PFS curve represents the cohort in the PF state. The area between the OS curve and the PFS curve represents the cohort with PD state. The area between the OS curve and the horizontal line of 100% represents the cohort in the D state (23). The model was run over a 20-year time horizon for the base case, which allowed for a follow-up of ≥ 99% of patients until death in the endocrine therapy group.

Results were expressed as total and incremental costs, lifeyears (LYs), quality-adjusted lifeyears (QALYs), and incremental cost-effectiveness ratios (ICERs). ICER was the main outcome measure and was expressed as the incremental cost per QALY gained. The PartSA was conducted using TreeAge Pro 2019.

#### Clinical Parameters

##### Efficacy

The PFS and OS data for ribociclib plus endocrine therapy and endocrine therapy alone were obtained from the phase III MONALEESA-7 study (13, 20). Fitted Weibull survival distributions as described above were used to populate the partitioned survival models.

##### Safety

The model included incidences and costs of two major grade 3/4 adverse events associated with the treatments as reported from the MONALEESA-7 clinical trial: neutropenia and leucopenia (**Table 1**) (13, 20).

**Table 1:** Model inputs.

Summary of Model Inputs				
Input	Value	Distribution	Note	Source
**Economic Input**				
Drug acquisition costs per 28-day of cycle, 2019 USD				
Tamoxifen	21.33	Gamma	WAC, pkg size: 30 of 20 mg	RED BOOK Online^®^ (24)
NSAI (letrozole or anastrozole)	7	Gamma	WAC, pkg size: 30 of 2.5 mg(letrozole), 30 for 1 mg (anastrozole)	RED BOOK Online^®^ (24)
Goserelin	635.25	Gamma	WAC, pkg size: 63 of 200 mg	RED BOOK Online^®^ (24)
Ribociclib	12,552.97	Gamma	WAC, pkg size: 63 of 200 mg	RED BOOK Online^®^ (24)
Disease management and monitoring costs per event, 2019 USD				
CT scan of chest	199	Gamma	1 for screening, every 8 weeks for 18 months and every 12 weeks after, and 1 for end of therapy	Physician Fee Schedule (CPT 71260) (25)
CT scan of abdomen and pelvis	324	Gamma	1 for screening, every 8 weeks for 18 months and every 12 weeks after, and 1 for end of therapy	Physician Fee Schedule (CPT 74177) (25)
Whole body bone scan	314	Gamma	1 for screening	Physician Fee Schedule (CPT 78306) (25)
Level 4 office visit (new)	131.18	Gamma	1 for screening	Physician Fee Schedule (CPT 99204) (25)
Level 4 office visit (established)	80.01	Gamma	2 for cycle 1, 1 for cycle 2, 2 for cycle3, 1 per cycle for subsequent cycles	Physician Fee Schedule (CPT 99214)
ECG (standard 12-lead)	8.65	Gamma	1 for screening, 2 for cycle 1 to cycle 3, 1 per cycle for subsequent cycles	Physician Fee Schedule (CPT 93010) (25)
Cardiac imaging (ECHO)	210.47	Gamma	1 for screening	Physician Fee Schedule (CPT 93306) (25)
Cardiac assessment (MUGA)	237.50	Gamma	1 for screening	Physician Fee Schedule (CPT 78472) (25)
CBC with auto diff WBC	8.63	Gamma	1 for screening, 2 for cycle 1 to cycle 3, 1 for each subsequent cycle after cycle3, 1 for end of therapy	Clinical Laboratory Fee Schedule (HCPCS 85025) (26)
CMP	11.74	Gamma	1 for screening, 2 for cycle 1 to cycle 3, 1 for each subsequent cycle after cycle3, 1 for end of therapy	Clinical Laboratory Fee Schedule (HCPCS 80053) (26)
Fasting lipid panel	14.88	Gamma	1 for cycle 1, 1 every 4^th^ cycle for each subsequent cycle after cycle 3, 1 for end of therapy	Clinical Laboratory Fee Schedule (HCPCS 80061) (26)
Free Assay (ft-3)	18.82	Gamma	1 for cycle 1, 1 every 4^th^ cycle for each subsequent cycle after cycle 3, 1 for end of therapy	Clinical Laboratory Fee Schedule (HCPCS 84481) (26)
TSH	18.67	Gamma	1 for cycle 1, 1 every 4^th^ cycle for each subsequent cycle after cycle 3, 1 for end of therapy	Clinical Laboratory Fee Schedule (HCPCS 84443) (26)
FSH	20.65	Gamma	1 for cycle 1, 1 every 4^th^ cycle for each subsequent cycle after cycle 3, 1 for end of therapy	Clinical Laboratory Fee Schedule (HCPCS 83001) (26)
Estradiol	31.04	Gamma	1 for cycle1, 1 for cycle 3	Clinical Laboratory Fee Schedule (HCPCS 82670) (26)
Prothrombin time/INR	4.37	Gamma	1 for screening, 1 for cycle 2, 1 for cycle 3, 1 for each subsequent cycle from cycle 3, 1 for EOT	Clinical Laboratory Fee Schedule (HCPCS 85610) (26)
Urinalysis	4.02	Gamma	1 for screening, 1 as clinically indicated during cycle 1 through subsequent cycles, 1 for EOT	Clinical Laboratory Fee Schedule (HCPCS 81000) (26)
Hepatic function panel	9.08	Gamma	1 for cycle 1,2,3, and 1 for cycle 4,5,6	Clinical Laboratory Fee Schedule (HCPCS 80076) (26)
Serum Pregnancy Test	16.73	Gamma	1 for screening, 1 for cycle 1, 1 for cycle 2, 1 for cycle 3, 1 for each subsequent cycle, 1 for EOT	Clinical Laboratory Fee Schedule (HCPCS 84702) (26)
Urine Pregnancy Test	8.61	Gamma	1 for screening, 1 for cycle 1, 1 for cycle 2, 1 for cycle 3, 1 for each subsequent cycle, 1 for EOT	Clinical Laboratory Fee Schedule (HCPCS 81025) (26)
Tumor tissue	273.00	Gamma	1 for screening, 1 for EOT	Clinical Laboratory Fee Schedule (HCPCS 86152) (26)
Blood for circulating tumor DNA	22.28	Gamma	1 for cycle 1, 1 for each subsequent cycle from cycle 8 day 1 and day 1 of every 3rd cycle thereafter, 1 for EOT	Clinical Laboratory Fee Schedule (HCPCS 87149) (26)
Blood test for CYP2D6	450.91	Gamma	1 cycle for cycle 1	Clinical Laboratory Fee Schedule (HCPCS 81226) (26)
Quantitative assay for serum drug level	18.64	Gamma	Tamoxifen-treated group: 1 for cycle 1, 2 for cycle 3 NSAI-treated group: 1 for cycle1, 1 for cycle 3	Clinical Laboratory Fee Schedule (HCPCS 80299) (26)
Cost of managing grade ¾ adverse events per episode, 2019 USD				
Neutropenia	9649.00	Gamma	We assumed grade ¾ adverse events occur during the first cycle of the treatment	Reference (27)
Leukopenia	4934.00	Gamma		Reference (28)
Cost of end-of-life-care, 2019 USD				
End-of-life care	20,409	Gamma	One time exit cost from progression-free and progressed disease state	Reference (29)
Subsequent therapy, 2019 USD				
Chemotherapy per month	5349.74	Gamma	Duration of therapy: 3.3 months	Reference (30, 31)
Hormone therapy per cycle	3687.86	Gamma	Drug acquisition cost and injection cost for the first 28 days	RED BOOK Online^®^ (24) Medicare Part B Drug Average Sales Price (HCPCS J9395) (32) Reference (31)
	1843.93	Gamma	Duration of therapy: 2.9 months Drug acquisition cost and injection cost after the first cycle (28 days)	
**Clinical inputs**				
AEs, probability, %				
Placebo group				
Neutropenia	63.5%	Beta		Reference (20)
Leukopenia	1.61%	Beta		Reference (20)
Ribociclib group				
Neutropenia	4.50%	Beta		Reference (20)
Leukopenia	1.80%	beta		Reference (20)
Patient receiving subsequent therapy, %				
Placebo group				
Chemotherapy	36.5%	Beta		Reference (20)
Hormone therapy	42.9%	Beta		Reference (20)
Ribociclib group				
Chemotherapy	30.6%	Beta		Reference (20)
Hormone therapy	44.8%	Beta		Reference (20)
Weibull distribution parameters				
Input	Scale	Shape	Note	Sources of original KM curves
Placebo group				
Progression-free survival	1.608	1.068		Figure 2A in Reference (13)
Overall survival	5.127	1.317		Figure 1A in Reference (20)
Ribociclib group				
Progression-free survival	2.593	1.146		Figure 2A in Reference (13)
Overall survival	7.710	1.107		Figure 1A in Reference (20)
**Humanistic outcome inputs**				
Input	Utility	Distribution	Note	Source
Health-state utility values				
Progression-free	0.830	Beta		Reference (33)
Progressed disease	0.443	Beta		Reference (34)
AE disutility values				
Neutropenia	-0.007	Beta		Reference (35)
Leukopenia	-0.003	Beta		Reference (35)

##### Quality of life

To evaluate health outcomes, literature-based utilities and disutilities were applied to the model by downwardly adjusting life years to generate QALYs. QALYs were estimated by assigning health state-specific utility values for advanced metastatic breast cancer for time spent within each health state as simulated by the partition model (33, 34). Disutilities associated with adverse events were applied to the proportion of patients experiencing that AE for one month for each AE episode based on incidences reported in the clinical trials (35).

#### Economic Parameters

##### Cost

Medical costs from a US healthcare payer perspective were considered in the model, which included drug costs, costs of disease monitoring and management, costs of management of severe adverse events (AEs), cost of subsequent therapy, and end-of-life costs. Drug acquisition costs were obtained from the RED BOOK using wholesale acquisition cost (WAC) prices. Costs of clinical laboratory tests were obtained from the Medicare Clinical Laboratory Fee Schedule, and costs of other health care services including office visits and imaging were obtained from the Medicare Physician Fee Schedule. Costs of treating severe AEs and end-of-life costs were estimated using published costs from the literature and applied as one-time events (**Table 1**). The base-case model assumed that AEs occurred during the first four weeks of initiating the therapies. All costs were standardized to 2019 U.S. dollars using the Consumer Price Index’s medical component and discounted by 3% annually (36).

##### Healthcare Resource Utilization

The drug utilization regimen, schedule for clinical lab tests, office visits, and imaging were modeled following the MONALEESA-7 clinical trial protocols per treatment cycle basis (20). Patients were assumed to be on treatment until disease progression. The types and probabilities of AEs were derived from the results of the MONALEESA-7 clinical trial and were modeled as a one-time event (20). Subsequent post-progression chemotherapy and hormone therapy were given to 36.5% and 42.9% of patients in the control arm and 30.6% and 44.8% of patients in the ribociclib, respectively (20).

### Sensitivity Analyses

#### One-Way Sensitivity Analysis

Deterministic one-way sensitivity analyses were conducted by varying one variable (model input) at a time within its plausible range. The plausible range was set to be plus or minus 25% of the base case value or 95% confidence interval (CI) except costs, which were set to be 50% to 200% of the base case value.

#### Probabilistic Sensitivity Analysis

Probabilistic sensitivity analysis was conducted by varying all variables at the same time by running 10,000 Monte Carlo simulations with probabilities and health state utility values set to follow beta distributions and costs set to follow gamma distributions. Cost-effectiveness acceptability curves were plotted based on the probabilistic sensitivity analysis.

#### Scenario Analyses

Additional scenario analyses were conducted. The analyses examined the model uncertainties around model structural variations by varying time horizons, (5-, 10-, 30-, 40-year time horizons), discount rate (0% or 3% for costs and QALYs), and excluding adverse events and subsequent therapies from the analysis.

## Results

### Base-Case Analysis Results

The base case cost-effectiveness results are summarized in **Table 2**. Within a 20-year time horizon, the partitioned survival analysis model predicted that ribociclib plus endocrine therapy was associated with 7.08 LYs and 4.09 QALYs as compared to 4.72 LYs and 2.70 QALYs in the endocrine therapy only arm. While the ribociclib group is associated with significantly longer LYs and QALYs, it is also associated with significantly higher costs. The expected annually discounted (3%) total costs for ribociclib plus endocrine therapy arm per patient were $446,130 as compared to $52,699 in the endocrine therapy only arm. The drug costs of $398,041 accounted for the most significant proportion of total costs followed by end-of-life care ($16,140), disease management and monitoring ($15,497), and subsequent therapy after progression ($9,528). Cost for ribociclib acquisition itself constituted 95% of total medication cost and 85% of total cost in the ribociclib and endocrine therapy group. As a result, the ICER for ribociclib plus endocrine therapy compared to the endocrine therapy alone is $166,689/LY and $282,996/QALY. At a willingness-to-pay (WTP) threshold of $150,000/QALY accepted in oncology treatments in the United States, this ribociclib treatment cannot be considered as a cost-effective option compared to endocrine therapy alone.

**Table 2:** Final/base case cost effectiveness analysis.

Base case cost-effectiveness analysis		
Base-case-results	Ribociclib	Placebo
Costs (USD)		
Medication	$398,042	$12,615
Disease management and monitoring	$9,528	$10,410
Managing adverse events	$15,497	$11,330
Subsequent therapy after progression	$6,923	$523
End-of-life care	$16,140	$17,821
** Total cost**	$446,130	$52,699
		
Effectiveness		
Progression-free life years	2.47	1.57
Post-progression life-years	4.61	3.15
**Total life years**	7.08	4.72
		
Progression-free QALY	2.04	1.30
Post-progression QALY	2.04	1.40
**Total QALY**	4.09	2.70
		
Incremental Cost-effectiveness		
Incremental cost per life-year gained ($/LY)	166,690	
Incremental cost per QALY gained ($/QALY)	282,996	

Ribociclib cost itself was $378,561, which is 95.11% of total medication cost and 84.85% of the total cost ($446,130) in the ribociclib group.

### Scenario Analysis Results

Additional scenario analyses were conducted to explore the effect of varying time horizons, discount rate, and the exclusion of AEs, and the exclusion of subsequent therapies from the base case analysis (**Table 3**).

**Table 3:** Results of scenario analyses.

Parameter	Base case input	Alternative input(s)	ICER ($/QALY)
Base-case result			282,996
Time horizon	20 years	5 years	831,552
		10 years	434,562
		30 years	260,519
		40 years	256,529
Discount rate	3% for cost only	3% for cost and benefits	358,418
		No discount rate applied for cost and benefit	301,841
Adverse event cost/disutilities	Included	Excluded	278,393
Subsequent therapies	Included	Excluded	283,631

#### Varying Time Horizons

With the base case time horizon set to be 20 years, the scenario analysis varied the time horizon at 5, 10, 30 and 40 years. These changes resulted in the ICER varying from $831,551/QALY (with incremental cost: $358,692 & incremental QALY: 0.43), $434,562/QALY (with incremental cost: $389,793 & incremental QALY: 0.90), $260,519/QALY (with incremental cost: $393,871 & incremental QALY: 1.51), and $255,805/QALY (with incremental cost: $393,939 & incremental QALY: 1.54), respectively. ICERs are relatively stable beyond 20 years of follow-up in comparison with the base case ICER of $282,996/QALY with 20 years of time horizon, with none reaching cost-effectiveness.

#### Varying Discount Rates

In the base-case analysis, a 3% discount rate was applied only to cost, while the effectiveness outcome of QALY was not discounted. When a 3% discount rate was applied to both cost and QALY, the ICER increased to $358,417/QALY. When no discounting was applied, the ICER was similarly above the WTP threshold ($301,841/QALY).

#### Exclusion of Adverse Events

The base-case model included adverse events for neutropenia and leukopenia and assumed that an adverse event occurred during the first four weeks of initiating the therapies. When cost and disutility information related to these adverse events were excluded, the ICER changed from $282,996/QALY to $278,393/QALY. Despite the prevalence of these AEs, they appeared to have little impact on both the QALYs (<0.5%) and costs (<2%).

#### Exclusion of Subsequent Therapy Costs

In the base-case model, patients received subsequent chemotherapy or endocrine hormone therapies once they discontinued the main assigned medication treatments. When subsequent therapy costs were excluded from the model, the ICER result changed from $282,996/QALY to $283,630/QALY, which showed this had a negligible impact on the model.

### One-Way Deterministic Sensitivity Analysis Results

**Figure 3:**
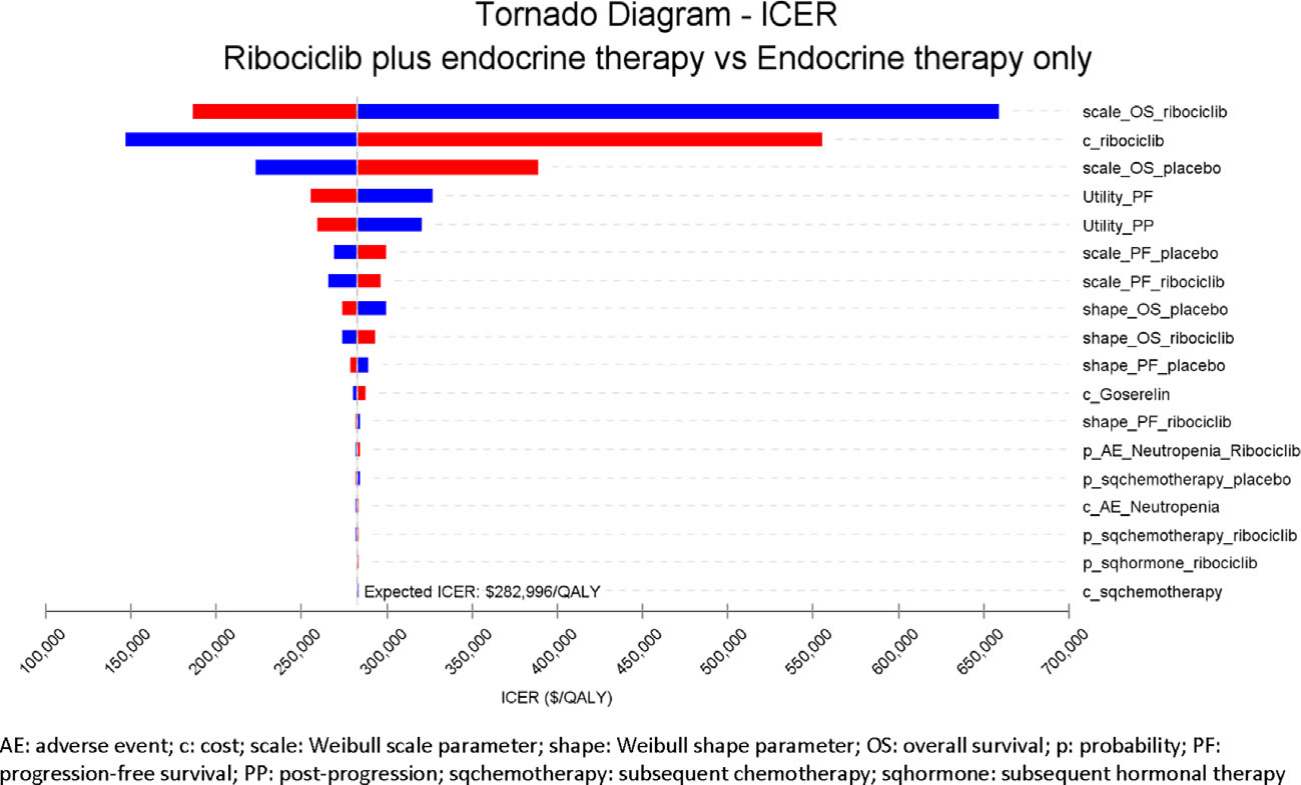
Tornado Diagram for One-way Deterministic Sensitivity Analysis.

The one-way sensitivity analysis results are shown in the tornado diagram in **Figure 3**, which lists the model inputs that have the biggest impact on the cost-effectiveness when individual model inputs were varied within their plausible ranges. The tornado diagram ranks the variability of each input parameter from the highest to the lowest across the top 18 model inputs based on their impact on the ICER. Key model drivers were scale parameters for OS Weibull distribution for both treatment arms as well as the drug cost of ribociclib. Overall, the model is robust with the ICER maintained above $150,000/QALY across all variations as validated by the one-way sensitivity analysis.

### Probabilistic Sensitivity Analysis Results

**Figure 4:**
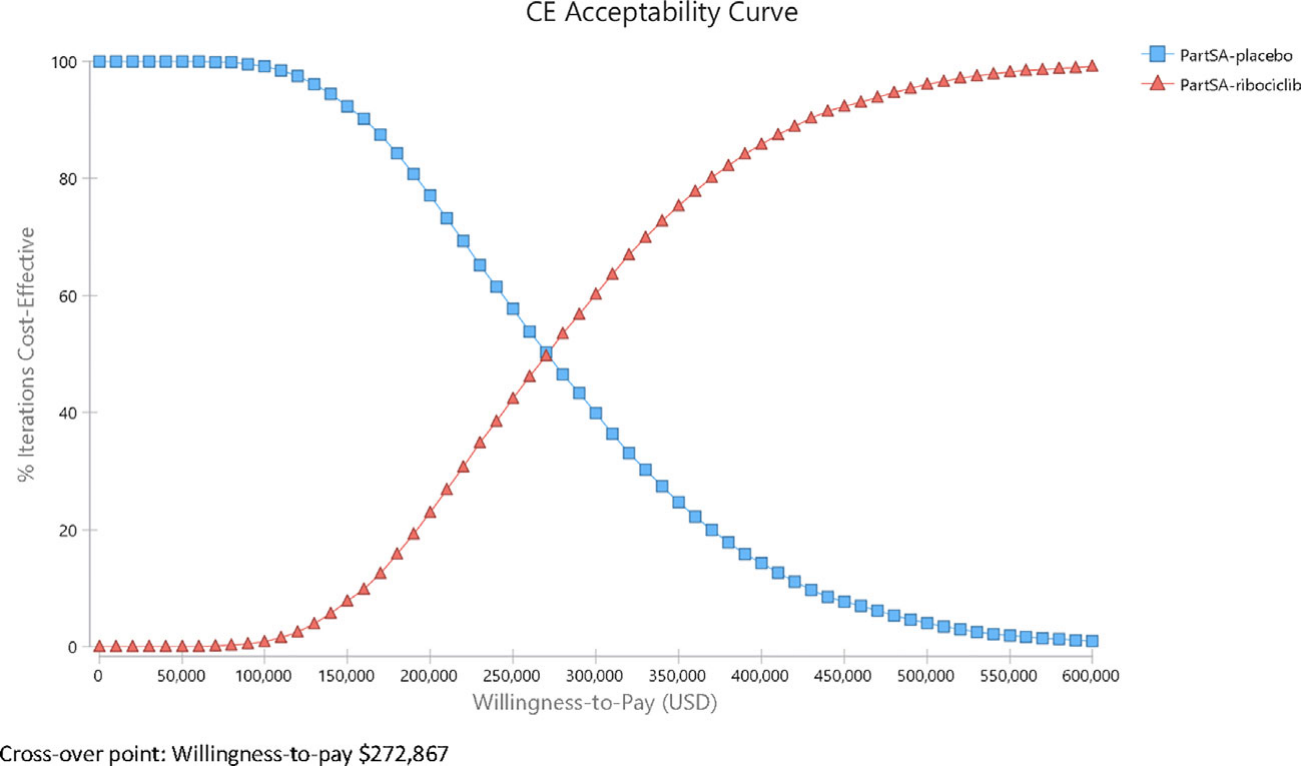
Cost-effectiveness Acceptability Curve.

A probabilistic sensitivity analysis using a Monte Carlo simulation of 10,000 iterations demonstrated that in most iterations (>99%), the ribociclib group yielded more QALYs and was more costly. The cost-effectiveness acceptability curves (CEAC) from the probabilistic sensitivity analysis (**Figure 4**) present the probabilities for each alternative that have the greatest net benefit (add up to 1 at any given WTP), expressed as a function of the WTP. The CEACs showed that ribociclib was less likely to be associated with more net benefit than placebo below a WTP of $272,867/QALY. Only above $272,867/QALY did ribociclib start to have an advantage over endocrine therapy. At a $100,000/QALY WTP threshold, the endocrine therapy is more cost-beneficial than ribociclib plus endocrine therapy in 99.12% of the iterations, and at a $150,000/QALY WTP threshold, in 92.91% of the iterations, showing the stability of the base-case results.

## Discussion

The approval of ribociclib for use in pre/perimenopausal women give patients access to a new treatment option that helps to improve progression-free survival and overall survival. Building on clinical trial data, this study provides a cost-effective evaluation of this new drug treatment. While significantly improving PFS and OS over endocrine therapy alone, ribociclib plus endocrine therapy for pre/perimenopausal women with HR+/HER2- advanced breast cancer is not cost-effective with an ICER of $282,996/QALY, despite assuming a generous WTP threshold of $150,000/QALY for the U.S. healthcare setting. As tested by different scenario, deterministic and probabilistic sensitivity analyses, the model is robust with the ICER remained above $150,000/QALY.

To the best of our knowledge, our study is the first study to use a partitioned survival analysis model to evaluate the cost-effectiveness of ribociclib for this indication in pre/perimenopausal women. Our findings showed that the drug price of ribociclib has one of the biggest impacts on the cost-effectiveness result. At an acquisition cost of $12,553 for 63 200 mg tablets for a 28-cycle treatment, ribociclib drug costs totaled $374,577, accounting for 84.9% of the total costs of the ribociclib treatment arm. In fact, the incremental cost of ribociclib plus endocrine therapy arm versus the endocrine therapy only arm was $393,431, most of which was contributed by ribociclib drug cost. The differentials in other cost categories, including disease monitoring, adverse event management, post-progression treatment and end-of-life costs are relatively low in comparison. Threshold analysis was conducted and shows that at a WTP of $150,000/QALY, the drug price needs reduction by about 48.84% (from $12,553 to $6,422 per 28-day treatment cycle for 63 200mg tablets) in order to make ribociclib a cost-effective treatment option in this population.

Our findings in the pre/perimenopausal patient population are in line with other studies evaluating cost-effectiveness of ribociclib plus endocrine therapy for HR+/HER2- advanced or metastatic breast cancer in the postmenopausal population, which also found the intervention to be not cost-effective with ICERs ranging from $210,369/QALY to $440,000 per QALY based on MONALEESA-2(NCT01958021) clinical trial (33, 37). It should be noted that MONALEESA-7 was different from the above trial in that it was designed for pre/perimenopausal women who also received ovarian function suppression (OFS) during treatment, yet in both patient populations, the high drug costs could not be justified by the received benefits at currently accepted WTP threshold from the US payer perspective.

The high drug costs also directly impact patients through high out-of-pocket costs which often place patients at risk of financial toxicity (38). The annual acquisition cost for ribociclib treatment is $163,189 in 2019 USD. Although patient out-of-pocket costs for ribociclib was not assessed in this study, it could be substantial even for insured patients given that ribociclib is typically listed as a specialty drug with formulary tier 4 or 5 with different insurance plans. The financial burden for patients and caregivers may lead to psychological distress and medication nonadherence, leading to diminished patient clinical and humanistic outcomes (38, 39).

Ribociclib is not alone in this regard. A recent study assessed the cost-effectiveness evaluations of cancer drugs conducted by the US’s Institute for Clinical and Economic Review (ICER), and found that most new cancer drugs were not cost-effective (40). In the US, regulatory approvals are not tied to costs and the complicated network of multiple payers in the healthcare system is associated with less negotiating power to obtain substantial drug discounts with manufacturers as compared to other countries with single-payer health care systems. In general, the US pays the highest price for new drugs in the world, especially for specialty drugs, including oncology drugs. For example, the US pays about 46% of global expenditure on oncology medications (41). While high drug prices help incentivize innovation, there needs to be a fine balance between profitability to the pharmaceutical industry and affordability to the health care systems (42). Cost-effectiveness evidence of new oncology drugs should be utilized to play a bigger role in guiding pricing and reimbursement decisions with US payers through novel payment models (43, 44).

This study has several strengths. First, the study applied a partitioned survival analysis (PSA) model to simulate the lifetime costs and QALYs among the patient population. Compared to traditional Markov models, PSA models directly estimate health state occupancy from the survival curves without needing to calculate transition probabilities needed for Markov models. Thus, PSA modeling is viewed as a relatively more straight-forward approach to make estimates from clinical trial data and tends to provide a better estimate to the observed survival data. PSA models are the predominantly used model structure in oncology treatments in health technology assessment (HTAs) submitted to (conducted by) the UK National Institute for Health and Care Excellence (NICE), which represented some of the highest standards for cost-effectiveness analysis. Second, the study extrapolated both progression-free and overall survivals beyond the trial period using fitted parametric curves to allow for evaluation of costs and outcomes over life time. Third, the study extensively evaluated the model uncertainties by including not only one-way and probabilistic sensitivity analyses but also different scenario analyses to capture different possible clinical and modelling scenarios.

This study has limitations. One limitation is related to the model structure uncertainty, which is inherent to all pharmacoeconomic modeling. Currently, the use of PF, PD, and D as model health states are widely adopted in oncology cost-effectiveness studies because they follow the primary endpoints of oncology clinical trials. While it is possible to test the model input uncertainties using deterministic and probabilistic sensitivity analyses, the uncertainties around the model structure are more challenging to test.

Another limitation is related to uncertainties around model inputs. This model is based on large RCT efficacy and safety data and may not necessarily reflect real-world outcomes and cost-effectiveness. For new drugs or existing drugs for new indications, initial cost-effectiveness assessment typically is conducted based on clinical trial data as not much real-world evidence is available yet. It will be desirable to also evaluate the cost-effectiveness when real-world effectiveness data become available. Besides, the utilities used in the model were not directly from the study population but from the literature on similar populations. However, we used the best possible data estimates from the literature. Also, as the clinical trial publications did not report detailed data on dose reduction and discontinuation, their impacts on the cost of treatment were not included in this model.

In conclusion, based on the partitioned survival analysis, this study found that while ribociclib provides significant survival benefits, it does not appear to be cost-effective when compared to endocrine therapy alone in treating pre/perimenopausal women with HR+/HER2- advanced breast cancer with a WTP of $150,000/QALY. Healthcare decision-makers need to take this into consideration, along with clinical effectiveness and safety when selecting treatments across populations to ensure efficient allocation of limited health care resources. Reductions in drug cost, could bring this treatment more in-line with accepted cost-effectiveness WTP limits across cancer treatments.

## Data Availability Statement

The datasets generated for this study are available on request to the corresponding author.

## Author Contributions

EJ and LZ conceived and designed the study. EJ and LZ performed data collection. EJ and LZ performed data analysis. EJ, CW, LW, and LZ wrote and revised the manuscript. All authors contributed to the article and approved the submitted version.

## Conflict of Interest

The authors declare that the research was conducted in the absence of any commercial or financial relationships that could be construed as a potential conflict of interest.
